# RIPK3 Contributes to Lyso-Gb3-Induced Podocyte Death

**DOI:** 10.3390/cells10020245

**Published:** 2021-01-27

**Authors:** So-Young Kim, Samel Park, Seong-Woo Lee, Ji-Hye Lee, Eun Soo Lee, Miri Kim, Youngjo Kim, Jeong Suk Kang, Choon Hee Chung, Jong-Seok Moon, Eun Young Lee

**Affiliations:** 1 Department of Internal Medicine, Soonchunhyang University Cheonan Hospital, Cheonan 31151, Korea; ksyoung801@gmail.com (S.-Y.K.); samelpark17@schmc.ac.kr (S.P.); lju600@naver.com (S.-W.L.); abalonea@naver.com (J.S.K.); 2 Department of Integrated Biomedical Science, Soonchunhyang Institute of Medi-Bio Science, Soonchunhyang University, Cheonan 31151, Korea; kimmiri0715@gmail.com (M.K.); yjokim@sch.ac.kr (Y.K.); 3 BK21 Four Project, College of Medicine, Soonchunhyang University, Cheonan 31151, Korea; 4 Department of Pathology, Soonchunhyang University Cheonan Hospital, Cheonan 31151, Korea; whui01@schmc.ac.kr; 5 Department of Internal Medicine, Yonsei University Wonju College of Medicine, Wonju 03722, Korea; es1121@yonsei.ac.kr (E.S.L.); cchung@yonsei.ac.kr (C.H.C.); 6 Institute of Tissue Regeneration, College of Medicine, Soonchunhyang University, Cheonan 31151, Korea

**Keywords:** Fabry disease, lyso-Gb3, alpha-galactosidase A, RIPK3, necroptosis, alpha-galactosidase

## Abstract

Fabry disease is a lysosomal storage disease with an X-linked heritage caused by absent or decreased activity of lysosomal enzymes named alpha-galactosidase A (α-gal A). Among the various manifestations of Fabry disease, Fabry nephropathy significantly affects patients’ morbidity and mortality. The cellular mechanisms of kidney damage have not been elusively described. Necroptosis is one of the programmed necrotic cell death pathways and is known to play many important roles in kidney injury. We investigated whether RIPK3, a protein phosphokinase with an important role in necroptosis, played a crucial role in the pathogenesis of Fabry nephropathy both in vitro and in vivo. The cell viability of podocytes decreased after lyso-Gb3 treatment in a dose-dependent manner, with increasing RIPK3 expression. Increased reactive oxygen species (ROS) generation after lyso-Gb3 treatment, which was alleviated by GSK’872 (a RIPK3 inhibitor), suggested a role of oxidative stress via a RIPK3-dependent pathway. Cytoskeleton rearrangement induced by lyso-Gb3 was normalized by the RIPK3 inhibitor. When mice were injected with lyso-Gb3, increased urine albuminuria, decreased podocyte counts in the glomeruli, and effaced foot processes were observed. Our results showed that lyso-Gb3 initiated albuminuria, a clinical manifestation of Fabry nephropathy, by podocyte loss and subsequent foot process effacement. These findings suggest a novel pathway in Fabry nephropathy.

## 1. Introduction

Fabry disease is a lysosomal storage disease with an X-linked heritage caused by absent or decreased activity of lysosomal enzymes named alpha-galactosidase A (α-gal A) [1,2]. Deficient or decreased activity of the enzyme leads to the progressive intracellular or lysosomal accumulation of globotriaosylceramide (Gb3) [3]. Although Fabry disease has serious clinical manifestations, including stroke, cardiomyopathy, renal failure, and death [4], the diagnosis of Fabry disease is challenging, especially in the early period of the disease [5]. Although the leading cause of death in patients with Fabry disease is cardiovascular disease, renal failure was the most common clinical event in male patients who died of cardiovascular disease [6]. The renal complications of Fabry disease, i.e., Fabry nephropathy, significantly affect patient’ morbidity and mortality, although it is clinically silent in the early course of the disease [7]. As a result, male patients with Fabry nephropathy reach end-stage kidney disease (ESKD) in the third to fifth decade of life [8]. When proteinuria is clinically prominent (>1 g/g) or the glomerular filtration rate (GFR) is already decreased, enzyme replacement therapy (ERT) cannot prevent the progressive loss of renal function [9,10].

The association between podocyte injury and Gb3 accumulation has been observed from the earlier phase of life in patients with Fabry disease [11]. Moreover, the inclusion of Gb-3 was observed in almost every glomerular cell type and was especially abundant in podocytes [11], suggesting that podocyte injury might be critical in the pathogenesis of Fabry nephropathy. Gb3 accumulation was associated with progressive podocyte injury and loss, increased foot process width, and foot process effacement [12,13]. The subsequent increase in urinary protein excretion is a risk factor for the progression of Fabry nephropathy [14]. Podocyturia could occur as an early marker even before the appearance of significant proteinuria, suggesting subclinical kidney injury [15]. Podocyte injury and the loss to Bowman’s space resulted in podocyte hypertrophy and the subsequent detachment from the glomerular basement membrane (GBM), leading to focal segmental glomerulosclerosis [16].

However, the cellular mechanisms of how kidney tissue becomes damaged in Fabry disease are unknown. The accumulation of Gb3 leads to kidney fibrosis [17,18,19]. A previous study reported that despite ERT, disease progression was not prevented, implying additional cellular pathways in addition to the intracellular accumulation of Gb3 contributed to renal failure in patients with Fabry nephropathy [20]. Globotriaosylsphingosine (lyso-Gb3) are circulating molecules, which, when observed in a high concentration in serum, indicated the presence of deacylated Gb3 in patients with Fabry disease [21]. Lyso-Gb3 was shown to induce epithelial-mesenchymal transition in kidney tubular epithelial cells via the PI3K/AKT pathway [22]. With the knock-out of GLA in podocytes, proliferation, pro-inflammatory processes, and cell death pathways were activated [23]. Although ERT with α-gal A cleared Gb3 deposition from podocytes, ERT did not reverse dysregulated autophagy, mTOR/AKT signaling, or a pro-fibrotic pathway in a culture model of human podocytes [24]. Although these previous studies tried to describe the pathogenesis of Fabry nephropathy at the molecular level, the mechanism of cell damage has not been fully elucidated.

Necroptosis, recently unveiled as a programmed cell death pathway morphologically featuring both necrosis and apoptosis, has been implicated in the development of several diseases [25,26]. Necroptosis is known to play many important roles in the pathway of kidney injury, especially in the setting of acute kidney injury (AKI) [27,28]. Receptor-interacting protein kinase 3 (RIPK3) plays a crucial role in the necroptosis pathway [26]. Although studies on the association between RIPK3 and chronic kidney disease (CKD) are lacking, we reported that RIPK3 played an important role in kidney fibrosis via the AKT pathway [29]. However, there has been no report on the association between RIPK3 and Fabry disease. Therefore, we investigated whether RIPK3, which is a protein phosphokinase with an important role in necroptosis, played a crucial role in the pathogenesis of Fabry nephropathy.

## 2. Materials and Methods

### 2.1. Podocyte Culture and Drug Treatment

Our study used conditionally immortalized mouse podocytes that were kindly provided by Dr. Peter Mundel [30,31]. The cells were incubated at two different temperature, at 33 °C under permissive conditions for proliferation in low glucose (5.5 mM) Dulbecco’s modified Eagles Medium (DMEM) supplemented with 10% fetal bovine serum (FBS), 10 U/mL mouse recombinant interferon-γ (IFN- γ, Sigma-Aldrich, St. Louis, MO, USA), and 1% penicillin-streptomycin, and at 37 °C for differentiation without IFN- γ for two weeks [30,31].

Lyso-Gb3 from porcine blood was purchased from Sigma-Aldrich. We dissolved the powder-form of lyso-Gb3 in dimethyl sulfoxide (DMSO) to generate a 2 mM stock and used it to treat immortalized mouse podocytes.

### 2.2. Measurement of Cytosolic ROS Generation

Cytosolic reactive oxygen species (ROS) generation was measured using 2′-7′ dichlorofluorescein diacetate (CM-H_2_DCF-DA, Invitrogen, Grand Island, NY, USA). Podocytes grown on a glass dish were loaded with 10 μM CM-H_2_DCF-DA for 30 min at 37 °C. Excess dye was washed out using 1× phosphate-buffered saline (PBS). The fluorescence intensity was measured using a Carl Zeiss LMS 710 confocal microscope.

### 2.3. Western Blotting

Cultured podocytes were homogenized in PRO-PREP^TM^ protein extraction solution (iNtRON Biotechnology, Seoul, Korea) containing a protease inhibitor cocktail (Roche Diagnostics GmbH, Mannheim, Germany). All samples were quantified using the Bradford assay (Bio-Rad, Hercules, CA, USA) with BSA as a standard, and an equal amount of each lysate was examined by sodium dodecyl sulfate-polyacrylamide gel electrophoresis (SDS-PAGE). The separated proteins were transferred to a polyvinylidene fluoride membrane (Millipore, Billerica, MA, USA). The membrane was blocked with 5% nonfat dry milk followed by primary antibody incubation at 4 °C overnight. Primary antibodies for RIPK3 (#AHP1797, rabbit, 1:1000, Bio-Rad) and β-actin (sc-4778, 1:2000, Santa Cruz Biotechnology, Santa Cruz, CA, USA) were prepared in 0.1% Tris-buffered saline containing Tween-20 and 1% milk at the appropriate dilution. Subsequently, the membranes were washed with PBS-Tween solution followed by incubation with horseradish peroxidase-conjugated secondary antibody. The bands were visualized with a ChemiDoc^TM^ XRS+ (Bio-Rad) imaging system using Luminata Forte enhanced chemiluminescence solution (Millipore).

### 2.4. Cell Cytotoxicity Assay

Cell cytotoxicity was measured from the culture medium of mouse podocytes using 3-(4,5-Dimethyl-2-thiazolyl)-2,5-diphenyl-2H-tetrazolium bromide (MTT) cytotoxicity assay (#M2128, Sigma-Aldrich) following the instructions from the manufacturer. Cells (0.4 × 10^5^ cells in 24-well plates for cell culture) were treated by lyso-Gb3, RIPK3 inhibitor, or both, then incubated for 24 h.

### 2.5. Transmission Electron Microscopy of Kidney

For the transmission electron microscopy (TEM) observations, the samples were fixed in 2.5% glutaraldehyde for 2 h at 4 °C, washed with 0.1 M phosphate buffer at pH 7.4, and then fixed in 1% osmium tetroxide for 90 min. The samples were dehydrated with a graded series of ethanol, exchanged in propylene oxide, and embedded with a mixture of Epon. Electron micrographs of each sample were taken at 20,000× magnification.

### 2.6. Immunofluorescence

Podocytes were grown on collagen-coated coverslips for 14 days, fixed with 4% paraformaldehyde (PFA), permeabilized with 0.25% Triton X-100, blocked with 1% BSA, incubated with primary antibodies for 1 h at 4 °C, and finally incubated with secondary antibodies for 1 h at room temperature. Primary antibodies against FITC-phalloidin (#P5282, 1:500, Sigma-Aldrich), synaptopodin (#163 002, rabbit, 1:1000, Synaptic Systems, Gottingen, Germany), and RIPK3 (#AHP1797, rabbit, 1:1000, Bio-Rad) were used. The images were collected using an LSM 710 confocal microscope (Carl Zeiss Microimaging, Thornwood, NY, USA). To quantitatively analyze the podocyte actin cytoskeleton, we conducted line region of interest (ROI) analysis of the fluorescence intensity for each picture and quantified the results using the ImageJ program (National Institutes of Health, Bethesda, MD, USA). Briefly, a line was drawn through the middle of the cell, then the pixel intensity along the line was measured using “plot profile” function in ImageJ. Next, we calculated area under the curve.

### 2.7. Animal Model

All mouse experimental protocols were approved by the Institutional Animal Care and Use Committee of Soonchunhyang University Asan, Korea (protocol #: SCH18-0041), and the methods were performed in accordance with the relevant guidelines and regulations. Twelve-week-old wild-type female C57/BL6 mice were used in this study. To generate human-mimicking Fabry mice, the mice received a single intraperitoneal injection of lyso-Gb3, which was adjusted to be the concentration of 100 nM in extracellular space, as previously reported [18]. The mice were euthanized 24 h after treatment with lyso-Gb3. Murine urine was collected four hours before sacrifice using metabolic cages. Urinary albumin (Exocell NephratII; Exocell Inc., Philadelphia, PA, USA) and creatinine (The Creatinine Companion; Exocell Inc.) levels were measured using ELISA kits.

For immunohistochemical staining, paraffin-embedded kidney samples were sliced into 4-µm-thick sections. The deparaffinized samples were incubated with anti-p57 (#57P06, 1:500, Thermo Scientific, Waltham, MA, USA) at 4 °C overnight. After washing three times, the samples were incubated with anti-rabbit IgG (#31460, 1:2000, Thermo Scientific) secondary antibody at room temperature for 30 min. Histologic changes were observed under a microscope (Olympus, Tokyo, Japan) and p57-staining was quantitated using ImageJ. To evaluate the p57-stained podocyte count, we randomly selected 30 glomeruli per mouse. Three mice were used in each group for podocyte counting.

### 2.8. Statistical Analysis

All analyses were performed using Prism version 8 (GraphPad Software, San Diego, CA, USA). The experimental values are presented as the mean ± SEM and SD. Statistical comparisons were made by the Mann-Whitney test, the Kruskal-Wallis test, one-way or two-way ANOVA, as appropriate. *p*-values of < 0.05 were considered significant.

## 3. Results

### 3.1. Lyso-Gb3 Induces RIPK3-Mediated Cell Death in Podocytes

**Figure 1:**
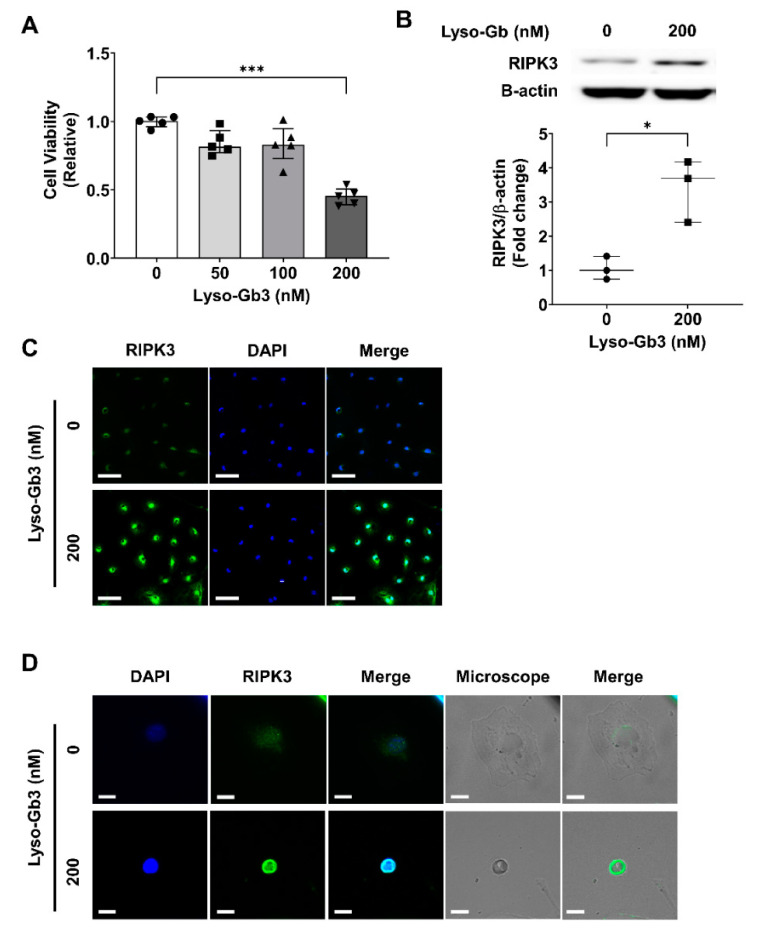
Lyso-Gb3 induced podocyte death via a RIPK3-dependent pathway in mouse podocytes. (**A**) MTT assay for cell viability. Mouse podocytes were treated with lyso-Gb3 for 24 h. Cell viability was reduced significantly in a dose-dependent fashion (*N* = 5, median with interquartile range). *** *p* < 0.001 versus control by the Kruskal–Wallis test with Dunn’s post-hoc analysis. (**B**) Representative Western blot assay (**top**) and densitometric quantification of RIPK3 levels (normalized to β-actin levels) (**bottom**). RIPK3 levels were increased by treatment of Lyso-Gb3 (200 nM) in mouse podocytes (*N* = 3, median with interquartile range). * *p* < 0.05 versus control using the Mann–Whitney test. (**C**,**D**) Immunofluorescence staining for RIPK3 (green) and DAPI (blue). (**C**) Representative images of RIPK3 expression (green) and DAPI (blue) in podocytes. The number of cells stained with RIPK3 was increased by lyso-Gb3 (200 nM) treatment. Magnification, 20×; Scale bars, 200 μm. (**D**) Immunofluorescence images obtained with an optical microscope showed that lyso-Gb3 induced cell shrinkage and the expression of RIPK3 in the cytosol. Magnification, 40×; Scale bars, 50 μm.

We treated podocytes with lyso-Gb3 in vitro to investigate whether lyso-Gb3 treatment resulted in podocyte death, representing the Fabry nephropathy model. First, we tested the effect of lyso-Gb3 on the viability of podocytes. The viability of podocytes was decreased after lyso-Gb3 treatment in a dose-dependent manner (Figure 1A). Since we previously showed the role of RIPK3 in the pathogenesis of CKD via the AKT pathway [29], the role of RIPK3 in the pathogenesis of podocyte death induced by lyso-Gb3 treatment was investigated. Lyso-Gb3 treatment increased RIPK3 protein levels (Figure 1B). The increased expression of RIPK3 in the cytosol was also confirmed through the immunofluorescence (IF) study (Figure 1C,D). Cell shrinkage was also observed after lyso-Gb3 treatment (Figure 1D), suggesting changes in the actin cytoskeleton in the mouse podocytes. Taken together, the results suggested that lyso-Gb3 treatment might lead to podocyte death via a RIPK3-dependent pathway.

### 3.2. Inhibition of RIPK3 Activity Suppresses Lyso-Gb3-Induced ROS Production

**Figure 2:**
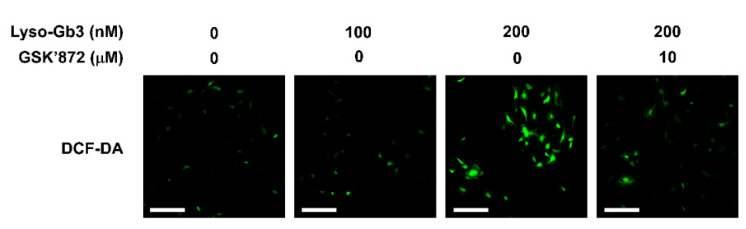
Association of lyso-Gb3 with ROS generation. Lyso-Gb3 treatment increased ROS production in a dose-dependent manner. GSK’872 treatment significantly reduced lyso-Gb3-induced ROS. Representative fluorescence images of lyso-Gb3-induced podocytes stained with 2′,7′ dichlorofluorescein (DCF-DA). Magnification, 20×; bar = 200 μM.

To explore the effect of lyso-Gb3 on RIPK3-dependent oxidative stress, podocytes were treated with lyso-Gb3 with or without GSK’872 for 24 h. DCF-DA staining was used to evaluate the degree of ROS production. ROS production was increased by lyso-Gb3 treatment in a dose-dependent manner (Figure 2). The generation of ROS by lyso-Gb3 was alleviated by GSK’872 treatment, suggesting that lyso-Gb3 induced oxidative stress via a RIPK3-dependent pathway.

### 3.3. Inhibition of RIPK3 Activity Suppresses Lyso-Gb3-Induced Cytoskeleton Impairment

**Figure 3:**
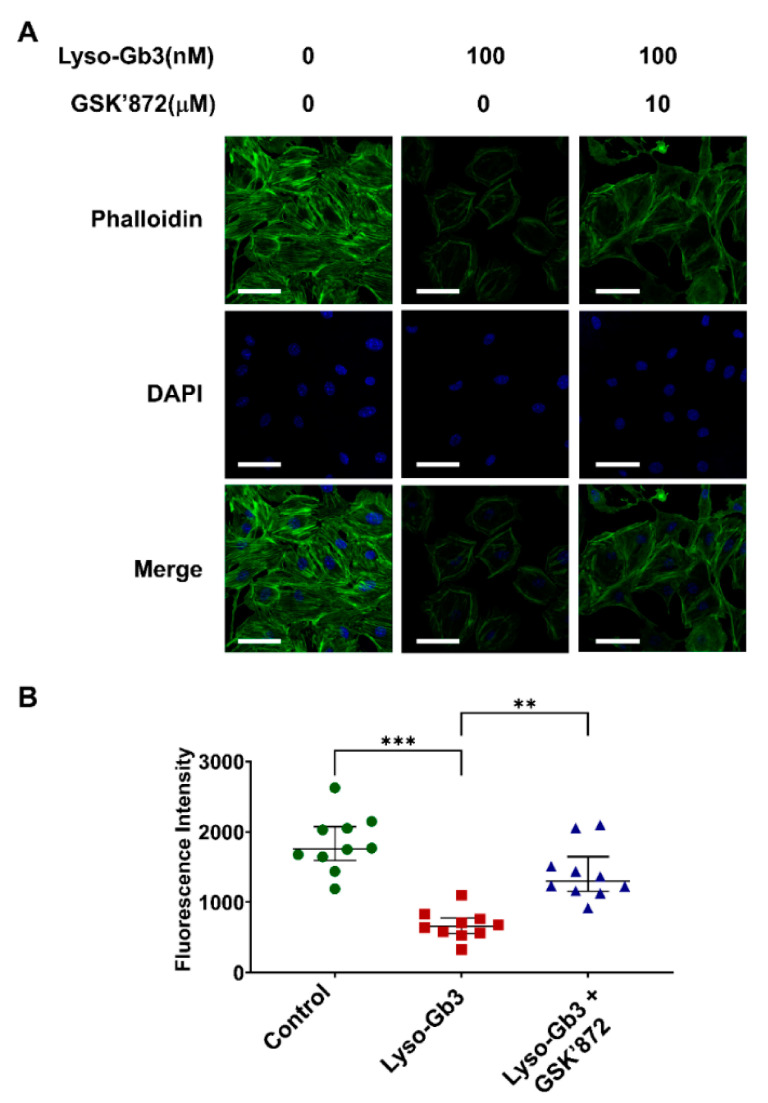
Cytoskeleton rearrangement induced by lyso-Gb3. Mouse podocytes grown on coverslips were treated with lyso-Gb3 with or without GSK’872 treatment. The cells were fixed with 4% PFA and immunolabeled with FITC-phalloidin (green) and DAPI (blue). (**A**) Representative morphologic changes in the actin cytoskeleton. Podocytes treated with lyso-Gb3 show in peripheral rearrangement of actin stained with phalloidin. The cytoskeleton arrangement was attenuated by GSK’872 treatment. Magnification, 40×; bar = 50 μm. (**B**) Quantitative analysis of fluorescence intensity of actin cytoskeleton rearrangement. The decreased fluorescence intensity in lyso-Gb3 treated podocytes was recovered by GSK’872 treatment (*N* = 10, median with interquartile range). ** *p* < 0.01, *** *p* < 0.001 by Kruskal-Wallis with Dunn’s post-hoc analysis.

Figure 3 shows a dot graph reflecting the quantified decrease in F-actin staining induced by treating mouse podocytes with lyso-Gb3. We observed that GSK’872 attenuated the cytoskeleton rearrangement induced by lyso-Gb3 (Figure 3), suggesting that lyso-Gb3 induced podocyte injury by actin cytoskeleton rearrangement through a RIPK3-dependent pathway.

### 3.4. Inhibition of RIPK3 Activity Suppresses Lyso-Gb3-Induced Cytotoxicity

**Figure 4:**
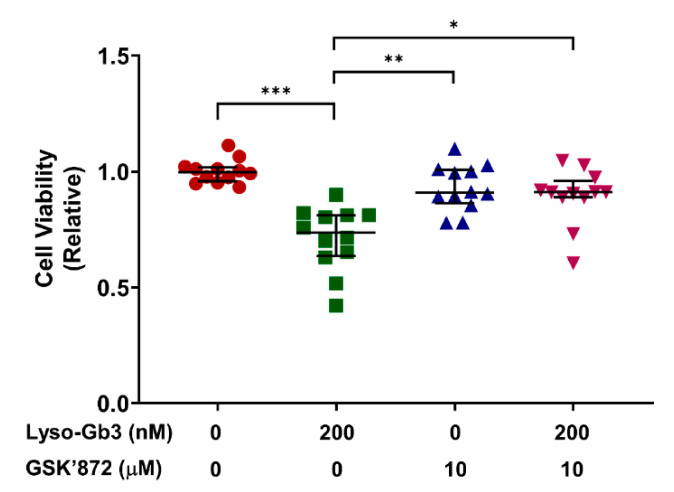
Cell viability after treatment with lyso-Gb3, GSK’872, or both. The leftmost group is the control group (neither lyso-Gb3 nor GSK’872). Each group was compared with every other group. Lyso-Gb3 (200 nM) treatment decreased cell viability. Cell viability improved after GSK’872 (10 μM) treatment (*N* = 12, median with interquartile range). * *p* < 0.05, ** *p* < 0.01, *** *p* < 0.001, by Kruskal-Wallis with Dunn’s post-hoc analysis.

Cell viability was evaluated using the MTT assay. Lyso-Gb3 induced cell death and GSK’872 alleviated it (Figure 4). Our in vitro model provided evidence that lyso-Gb3 activated a RIPK3-dependent cell death pathway, shown by increased RIPK3 and cell death after lyso-Gb3 treatment of mouse podocytes, and attenuation by GSK’872, a RIPK3 inhibitor. Next, we tried to validate these findings in an animal model.

### 3.5. RIPK3 Contributes to Lyso-Gb3-Induced Podocyte Injury In Vivo

**Figure 5:**
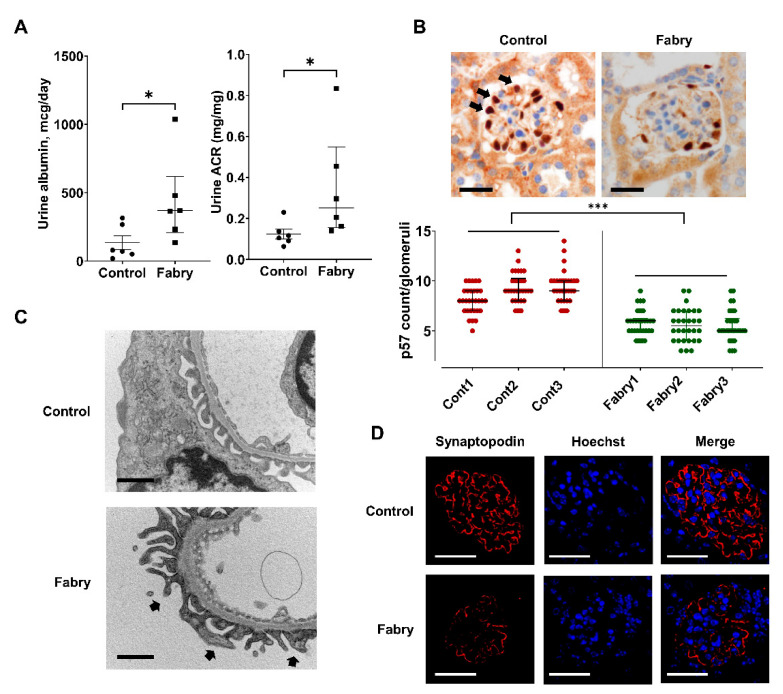
In vivo model of mouse treated with a single intraperitoneal lyso-Gb3 injection. (**A**) Albuminuria increased after lyso-Gb3 injection, suggesting lyso-Gb3 induced injury to the glomerular filtration barriers (*N* = 6, median with interquartile range, * *p* < 0.05, by Mann–Whitney test). (**B**) Representative histological images of lyso-Gb3-treated and control mice. p57 stain (black arrow) was used to identify podocytes. The number of p57 stained cells per glomeruli was reduced in mice injected with lyso-Gb3. Magnification, 40×; bar = 20 μm (three mice per group, 30 glomeruli per mouse, median with interquartile range, *** *p* < 0.001, by two-way ANOVA). (**C**) Representative TEM images of lyso-Gb3-treated and control mice. The control showed normal foot processes, whereas the mouse model mimicking Fabry disease showed partial segmental foot process effacement (black arrow). Magnification, 20,000×; bar = 1 μm. (**D**) IF showed decreased synaptopodin staining (red) in the glomerulus of a mouse injected with lyso-Gb3. Magnification, 40×; bar = 20 μm.

We injected mice with lyso-Gb3 to mimic Fabry nephropathy in mice [18], as mentioned above. Albuminuria increased after a single lyso-Gb3 injection (Figure 5A). In three mice in each group, p57-stained podocytes were counted in randomly selected 30 glomeruli per mouse, showed decreased podocyte counts in the mice glomeruli in the Fabry group (Figure 5B). TEM showed foot process effacement in the mice that received lyso-Gb3 (Figure 5C). Reduced synaptopodin staining implied that albuminuria was caused by podocyte loss (Figure 5D) and subsequent foot process effacement (Figure 5C).

## 4. Discussion

Our study showed both that lyso-Gb3 elicited cell death and that cell death might be through a RIPK3-dependent pathway in podocytes. Lyso-Gb3 are soluble circulating molecules [21], which have been considered an unidentified substance that proliferates vascular smooth muscle cells and cardiomyocytes in patients with Fabry disease [32]. Given that the intraperitoneal injection of lyso-Gb3 to mice led to inflammation seen in kidney histology [18], we hypothesized that circulating lyso-Gb3 could move to other organs to elicit disease phenotypes and observed that a single intraperitoneal injection of lyso-Gb3 induced the renal phenotype of Fabry disease. This not only suggested that the lyso-Gb3-attributed cell toxicity observed in in vitro studies [18,22] could be reproduced in animal models but also demonstrated that renal injury was initiated in the very early age of their life [7,11]. A single injection of an amount equivalent to the concentration of lyso-Gb3 in classical Fabry patients (about 100 nM) resulted in significant podocyte loss and segmental foot process effacement.

Necroptosis is a type of programmed necrotic cell death morphologically featuring both necrosis and apoptosis dependent upon RIPK3 and is implicated in the pathogenesis of several diseases, including myocardial infarction, stroke, atherosclerosis, AKI, sepsis, pancreatitis, and other several clinically important disorders [25,26]. Recent reports have shown the role in the pathogenesis of CKD, kidney fibrosis [29], and AKI to CKD transition [33]. Among the lysosomal storage diseases, RIPK3 was shown to have a role in the pathogenesis of Gaucher’s disease and Niemann–Pick disease [34,35]. However, the role of RIPK3 was not elucidated in Fabry disease. We first showed the role of RIPK3 in the pathogenesis of Fabry disease.

Strong evidence has advocated that necroptosis is involved in both the initiation and amplification of inflammatory responses in not only acute critical illness but also chronic inflammatory diseases [36]. Besides the role of RIPK3 in necroptosis, RIPK3 has a role in ROS generation by increasing aerobic respiration [37]. Previous studies have indicated that lyso-Gb3 induced oxidative damage to DNA in cultured human embryonic kidney cells [38] and that oxidative stress had a critical role in the pathogenesis of Fabry disease [39,40]. We showed that lyso-Gb3 induced ROS production and that the RIPK3 inhibitor, GSK’872, alleviated it, i.e., that lyso-Gb3-induced ROS generation was dependent upon RIPK3. Besides, lyso-Gb3 induced shrinkage of the podocytes (Figure 1D). Oxidative stress induces not only cell death but also a rearrangement of the actin cytoskeleton [41]. Similarly, we previously showed that blocking ROS generation restored actin cytoskeleton rearrangement [30]. Lyso-Gb3 treatment of podocytes induced the rearrangement of F-actin in the podocytes stained by phalloidin, and it was alleviated by RIPK3 inhibitor, suggesting that the actin cytoskeleton rearrangement induced by lyso-Gb3 relied upon a RIPK3-dependent pathway (Figure 3).

The actin cytoskeleton of podocytes is interconnected and faces mechanical forces from pulsatile blood flow and glomerular filtration, and the cytoskeletal dynamics should be regulated to preserve their structure to withstand shear stress and stretch in the glomeruli [42]. When podocytes fail to respond to such mechanical stress, podocyte dysfunction characterized by podocyte effacement and detachment could be observed as initial events. This process is known as the pathogenesis of focal segmental glomerulosclerosis (FSGS) [16,43]. Therefore, podocyte injury and subsequent podocyte detachment, i.e., podocyturia, might be the initial pathogenic events in Fabry nephropathy [11,15]. Although focal foot process effacement has been noted in early Fabry patients with normoalbuminuria [44,45], the effect of lyso-Gb3 on integrin and the slit diaphragm of podocytes is not known. A case report showed that a child without albuminuria had partially effaced foot processes and irregularly expressed nephrin [46]. In the present study, we observed that lyso-Gb3 induced actin cytoskeleton rearrangement (Figure 3), podocyte death (Figure 4), and increased albuminuria both with podocyte depletion and partial foot process effacement.

Our study had several limitations. First, we did not use an α-gal A knock-out mouse, since it was noted that, in α-gal A knock-out mice, Gb3 was accumulated in their organs without the phenotype of Fabry nephropathy [47]. Sanchez-Nino et al. showed that the lyso-Gb3 injection could be an alternative model mimicking Fabry nephropathy in mice [18]. Second, we did not measure Gb3 and lyso-Gb3 levels in mice plasma, since there is no commercially available ELISA kit to measure in murine plasma. Third, podocyte loss could not be quantified to the degree of whether it was attributed to either podocyte detachment by mechanical stress or podocyte death. Fourth, we used mouse podocyte. However, mouse podocytes were generally and broadly used to study diseases associated with podocytes. Fifth, RIPK3 expression could not be confirmed in human tissue because we did not have human kidney samples of patients with Fabry disease. Sixth, the detached urinary podocytes by a single injection of Lyso-Gb3 were not evaluated using specific stainings, such as synaptopodin, podocalyxin, urokinase-type plasminogen activator receptor, and CD80.

## 5. Conclusions

Despite the limitations, our results showed that lyso-Gb3 initiated Fabry nephropathy by podocyte loss and subsequent foot process effacement, leading to albuminuria via RIPK3 dependent pathway. Although, the results of this study should be confirmed in human histology, suggesting a novel pathway in Fabry nephropathy.
